# Supplementary material for: Allosteric regulation and crystallographic fragment screening of SARS-CoV-2 NSP15 endoribonuclease
Source: Nucleic Acids Res. 2023 Apr 28;51(10):5255–70. doi: 10.1093/nar/gkad314 (PMC10250223; doi:10.1093/nar/gkad314)
Supplement: gkad314_Supplemental_Files [file gkad314_supplemental_files.zip › Supplementary datasets.zip!/134/Refine_9/01-BUSTER/Cycle-1/shell.01/R_Rfree.html]

# Rxpct factors in resolution bins at cycle 0


|  |  |  |  |  |  |  |  |  |
| --- | --- | --- | --- | --- | --- | --- | --- | --- |
|  | **<d> (Å)**  Rxpct\_Work | Rxpct\_Free | Rxpct\_All | |  |  |  |  |
|  | 15.4464 | 0.3604 | 0.5434 | 0.3674 |  |
|  | 8.9180 | 0.2179 | 0.2605 | 0.2205 |  |
|  | 6.9078 | 0.2163 | 0.2586 | 0.2187 |  |
|  | 5.8382 | 0.2178 | 0.2919 | 0.2203 |  |
|  | 5.1488 | 0.1877 | 0.2491 | 0.1910 |  |
|  | 4.6573 | 0.1746 | 0.2324 | 0.1779 |  |
|  | 4.2841 | 0.1758 | 0.2058 | 0.1774 |  |
|  | 3.9882 | 0.1753 | 0.2301 | 0.1778 |  |
|  | 3.7463 | 0.1818 | 0.2442 | 0.1847 |  |
|  | 3.5436 | 0.1826 | 0.2350 | 0.1854 |  |
|  | 3.3707 | 0.1878 | 0.2197 | 0.1895 |  |
|  | 3.2208 | 0.2009 | 0.2388 | 0.2029 |  |
|  | 3.0893 | 0.2127 | 0.2233 | 0.2132 |  |
|  | 2.9727 | 0.2102 | 0.2240 | 0.2107 |  |
|  | 2.8683 | 0.2130 | 0.2345 | 0.2141 |  |
|  | 2.7743 | 0.2180 | 0.2582 | 0.2199 |  |
|  | 2.6889 | 0.2149 | 0.2125 | 0.2148 |  |
|  | 2.6109 | 0.2236 | 0.2277 | 0.2238 |  |
|  | 2.5394 | 0.2364 | 0.2518 | 0.2374 |  |
|  | 2.4734 | 0.2611 | 0.2543 | 0.2607 |  |
|  | **Overall** | 0.2043 | 0.2475 | 0.2065 |  |

# Rxpct factors in resolution bins at cycle 1


|  |  |  |  |  |  |  |  |  |
| --- | --- | --- | --- | --- | --- | --- | --- | --- |
|  | **<d> (Å)**  Rxpct\_Work | Rxpct\_Free | Rxpct\_All | |  |  |  |  |
|  | 15.4464 | 0.3596 | 0.5415 | 0.3665 |  |
|  | 8.9180 | 0.2177 | 0.2601 | 0.2202 |  |
|  | 6.9078 | 0.2150 | 0.2609 | 0.2176 |  |
|  | 5.8382 | 0.2162 | 0.2891 | 0.2187 |  |
|  | 5.1488 | 0.1857 | 0.2470 | 0.1890 |  |
|  | 4.6573 | 0.1712 | 0.2299 | 0.1745 |  |
|  | 4.2841 | 0.1719 | 0.2105 | 0.1740 |  |
|  | 3.9882 | 0.1728 | 0.2277 | 0.1754 |  |
|  | 3.7463 | 0.1786 | 0.2418 | 0.1816 |  |
|  | 3.5436 | 0.1807 | 0.2318 | 0.1834 |  |
|  | 3.3707 | 0.1855 | 0.2166 | 0.1872 |  |
|  | 3.2208 | 0.1985 | 0.2417 | 0.2009 |  |
|  | 3.0893 | 0.2114 | 0.2267 | 0.2122 |  |
|  | 2.9727 | 0.2097 | 0.2220 | 0.2101 |  |
|  | 2.8683 | 0.2140 | 0.2325 | 0.2149 |  |
|  | 2.7743 | 0.2165 | 0.2593 | 0.2185 |  |
|  | 2.6889 | 0.2157 | 0.2174 | 0.2158 |  |
|  | 2.6109 | 0.2221 | 0.2295 | 0.2225 |  |
|  | 2.5394 | 0.2355 | 0.2563 | 0.2368 |  |
|  | 2.4734 | 0.2615 | 0.2710 | 0.2620 |  |
|  | **Overall** | 0.2025 | 0.2474 | 0.2048 |  |

# Rxpct factors in resolution bins at cycle 2


|  |  |  |  |  |  |  |  |  |
| --- | --- | --- | --- | --- | --- | --- | --- | --- |
|  | **<d> (Å)**  Rxpct\_Work | Rxpct\_Free | Rxpct\_All | |  |  |  |  |
|  | 15.4464 | 0.3814 | 0.5637 | 0.3883 |  |
|  | 8.9180 | 0.2301 | 0.2510 | 0.2313 |  |
|  | 6.9078 | 0.2141 | 0.2484 | 0.2161 |  |
|  | 5.8382 | 0.2203 | 0.2773 | 0.2223 |  |
|  | 5.1488 | 0.1851 | 0.2657 | 0.1895 |  |
|  | 4.6573 | 0.1724 | 0.2417 | 0.1763 |  |
|  | 4.2841 | 0.1755 | 0.2295 | 0.1784 |  |
|  | 3.9882 | 0.1808 | 0.2387 | 0.1834 |  |
|  | 3.7463 | 0.1893 | 0.2395 | 0.1916 |  |
|  | 3.5436 | 0.1906 | 0.2394 | 0.1932 |  |
|  | 3.3707 | 0.1935 | 0.2160 | 0.1947 |  |
|  | 3.2208 | 0.2032 | 0.2338 | 0.2049 |  |
|  | 3.0893 | 0.2084 | 0.2508 | 0.2104 |  |
|  | 2.9727 | 0.2075 | 0.2210 | 0.2080 |  |
|  | 2.8683 | 0.2081 | 0.2251 | 0.2090 |  |
|  | 2.7743 | 0.2074 | 0.2427 | 0.2090 |  |
|  | 2.6889 | 0.2025 | 0.2086 | 0.2028 |  |
|  | 2.6109 | 0.2092 | 0.2163 | 0.2096 |  |
|  | 2.5394 | 0.2149 | 0.2229 | 0.2154 |  |
|  | 2.4734 | 0.2410 | 0.2403 | 0.2409 |  |
|  | **Overall** | 0.2054 | 0.2495 | 0.2077 |  |

# Rxpct factors in resolution bins at cycle 3


|  |  |  |  |  |  |  |  |  |
| --- | --- | --- | --- | --- | --- | --- | --- | --- |
|  | **<d> (Å)**  Rxpct\_Work | Rxpct\_Free | Rxpct\_All | |  |  |  |  |
|  | 15.4464 | 0.3597 | 0.5245 | 0.3660 |  |
|  | 8.9180 | 0.2184 | 0.2609 | 0.2209 |  |
|  | 6.9078 | 0.1972 | 0.2389 | 0.1996 |  |
|  | 5.8382 | 0.1907 | 0.2555 | 0.1929 |  |
|  | 5.1488 | 0.1597 | 0.2306 | 0.1635 |  |
|  | 4.6573 | 0.1481 | 0.2128 | 0.1517 |  |
|  | 4.2841 | 0.1524 | 0.2053 | 0.1553 |  |
|  | 3.9882 | 0.1565 | 0.2149 | 0.1592 |  |
|  | 3.7463 | 0.1658 | 0.2201 | 0.1683 |  |
|  | 3.5436 | 0.1671 | 0.2174 | 0.1698 |  |
|  | 3.3707 | 0.1735 | 0.2130 | 0.1757 |  |
|  | 3.2208 | 0.1856 | 0.2196 | 0.1874 |  |
|  | 3.0893 | 0.1949 | 0.2282 | 0.1965 |  |
|  | 2.9727 | 0.1956 | 0.2073 | 0.1960 |  |
|  | 2.8683 | 0.1986 | 0.2151 | 0.1995 |  |
|  | 2.7743 | 0.2004 | 0.2472 | 0.2025 |  |
|  | 2.6889 | 0.1995 | 0.2062 | 0.1998 |  |
|  | 2.6109 | 0.2072 | 0.2196 | 0.2079 |  |
|  | 2.5394 | 0.2190 | 0.2502 | 0.2210 |  |
|  | 2.4734 | 0.2397 | 0.2621 | 0.2410 |  |
|  | **Overall** | 0.1869 | 0.2343 | 0.1893 |  |
